# A New Deep Learning Methodology for Alarm Supervision in Marine Power Stations

**DOI:** 10.3390/s24216957

**Published:** 2024-10-30

**Authors:** José A. Orosa, Genaro Cao-Feijóo, Francisco J. Pérez-Castelo, José M. Pérez-Canosa

**Affiliations:** 1 Department of Navigation Sciences and Marine Engineering, University of A Coruña, Paseo de Ronda, 51, 15011 A Coruña, Spain; genaro.cao@udc.es (G.C.-F.); jose.pcanosa@udc.es (J.M.P.-C.); 2 Department of Industrial Engineering, University of A Coruña, 15405 A Coruña, Spain; francisco.javier.perez.castelo@udc.es

**Keywords:** control system, ships, CNN, power station, risk prevention

## Abstract

Marine engineering officers operate and maintain the ship’s machinery during normal navigation. Most accidents on board are related to human factors which, at the same time, are associated with the workload of the crew members and the working environment. The number of alarms is so high that, most of the time, instead of helping to prevent accidents, it causes more stress for crew members, which can result in accidents. Convolutional Neural Networks (CNNs) are being employed in the recognition of images, which depends on the quality of the images, the image recognition algorithm, and the very complex configuration of the neural network. This research study aims to develop a user-friendly image recognition tool that may act as a visual sensor of alarms adjusted to the particular needs of the ship operator. To achieve this, a marine engineering simulator was employed to develop an image recognition tool that advises marine engineering officers when they are conducting their maintenance activities, with the aim to reduce their stress as a work risk prevention tool. Results showed adequate accuracy for three-layer Convolutional Neural Networks and balanced data, and the use of external cameras stands out for user-friendly applications.

## 1. Introduction

Marine engineering officers are the officers of the engine room of ships. Their main competencies are associated with operating and maintaining marine machinery without power limits [1]. These operation and maintenance competencies are ensured by professional experience and, during their training studies, engine cadets are trained by engine room simulators [2] in accordance with maritime standards [3,4]. These engine room simulators are practically the same as the control panels employed in the engine control room. Engine room simulators, such as the one used in the present research, are widely used in maritime education and are supported in various ways by the IMO [5,6].

Once the cadets are trained in the operation and control of a power station of a ship by the simulators and with the computers of the engine control room, it is time for them to be trained in maintenance activities [7,8]. A clear difference between the engine room of a merchant ship and that of a navy ship is that, currently, in merchant ships, the maintenance of the equipment is carried out during navigation and there is only one engine room (in most navy ships, it is duplicated). As a consequence, the number of maintenance activities to be carried out in the hot and dangerous environment of the engine room highlights the need for any kind of help to control the power station (alarms and lights, among others) and, at the same time, to attend to the maintenance activities, reducing the stress of workers and its associated accidents due to human factors. To improve the maintenance asks, remote supervision is performed by software resources like AMOS Maintenance 7.0 [9]. Despite this, it is still not enough due to the reduced number of crew members on board.

At the same time, the engine room of a ship is a dangerous working place with several hazardous aspects, like loud noise (over 45 dB), high temperatures and relative humidity (over 40 °C) and vibration of the equipment. Electrical shocks with high-voltage equipment, steam burn in places near the boilers and many other accidents associated with the ship’s movement are only some examples of the risks involved. 

The engine room is considered by many authors as the “heart of the ship” [10] or the “core of a ship” in terms of power and electricity [11,12], so its permanent functioning is essential for ensuring the operation of all equipment on board and sea navigation. However, in spite of it being a department with several pieces of equipment of high difficulty and complexity, the number of crew members (even engineers) on board has been progressively reduced. For this reason, in recent years and using artificial intelligence and deep learning, the level of automation in the engine room has considerably increased, reaching the status of intelligent engine rooms [10,12]. For this mission, several alarms and monitoring systems have been implemented in a centralized control room of the ship’s engine room. Thus, it is possible that a reduced number of crew members can control and monitor the functioning of several pieces of equipment (auxiliary and main engines). However, in the normal functioning of the equipment on board, specifically when navigating in bad weather conditions, the engineers on duty have to face multiple alarms (visual and audible) that, in many cases, are not critical, triggering a stressful situation for the operator. In addition to this situation, it is relevant to keep in mind that the engine room is a strongly airtight and narrow space where workers spend a great deal of time during ocean sailing [13]. There, in many cases, the evolving conditions, such as weather conditions, workplace temperature, ship motion, noise and vibration, and workload and stress, are extreme [14].

For this reason, with the proposed method in the present paper, the operator can configure, with the aid of a simple video camera (visual sensor), a particular combination of visual alarms, which can detect a critical situation in some of the equipment, so that the engineers can find the cause of the failure and repair it promptly. With this proposed tool, the engineer on duty does not have to check all non-critical alarms, which are commonly raised during sea navigation. Then, considering that, as per the IMO, human factors are one of the key elements in maritime accidents [15], being responsible for about 80% of maritime accidents worldwide [16], the aim of the proposed method is to reduce the stress on crew members, increasing the overall safety of sea navigation and the efficiency for overall performance, which is particularly crucial for the reduced crew members on board actual ships. Furthermore, as the maintenance of machinery on board is carried out by the engineering officers and, according to the IMO’s accident investigation report, about 25% of maritime accidents are initially due to machinery failure [14], all external aids available to the officer on duty to reduce workload and stress can improve the overall safety of maritime transport.

The use of computer vision technology to monitor and recognize the engine room equipment was previously investigated by other authors [10]. Similar to the methodology followed in the present paper, artificial intelligence was previously used by other researchers to improve safety in the engine room. As early as 1992, some authors [17] proposed a prototype of intelligent monitoring to assist control center operators, in this case, a supervisor for a turbo-charger system of the ship’s main engine. Zou et al. [13] proposed a machine vision model (CWC-YOLOvs5) to identify early fires through smoke detection methods, replacing the standard convolution layer of the baseline model with coordconv (coordinate convolution) layers. Zhang et al. [18] proposed a proactive machine vision model based on the fusion of the transfer learning method and proactive perception technology for smoke detection. Qi et al. [19] also presented an auxiliary equipment detector in the cabin based on a deep learning model using the visual identification of auxiliary equipment in order to detect any anomalies. Therefore, in the literature, it is assumed that in intelligent engine rooms, as a part of intelligent ships, visual recognition is an essential technique for automatic inspection. Furthermore, initial problems, such as missing detection, low accuracy, slow speed and imperfect datasets, were addressed by researchers such as Shang et al. [20], who proposed a marine engine room equipment recognition model based on the improved You Only Look Once v5 (YOLOv5) algorithm. In the field of autonomous ships, some research is focused on fully intelligent and unmanned engine rooms, where the engine failure prediction and diagnosis are carried out using autonomous mobile robots [21].

Therefore, the use of artificial intelligence, big data and fully sensing the status of engine room equipment, which will gradually replace the naked eye and human labor, seems to be the direction of present and future developments, until the prospect of intelligent engine room systems can be reached [12].

Previous studies showed that the most relevant variable at the time to identify the causes of an accident based on CIAIN reports [22] is the human factor. The human factor is defined as the job factors that influence the behavior at work on board and that depend on the particularities of each crew member. In this sense, at the time of analyzing an accident, several variables probably related to accidents are defined, like visibility, number of crew members, ship dimensions, sea and wind conditions at the time of an accident and other variables [23]. Previous works revealed that the minimum number of crew members is associated with the human factor, which is a subjective variable too complicated to be controlled except, for instance, by increasing the minimum number of crew members on board [24]. Despite this, due to economic considerations, each ship owner tends to reduce the number of crew members meaning that artificial intelligence and more developed control systems are the only way to compensate for this loss of the human factor.

To improve the activities on board, on the main deck by deck officers, on the bridge by the officers on watch, or in engine room operations, recent machine learning research works are being developed [25,26]. Due to the fact that marine engineering control panels are designed in general terms to control a power station, they must never be modified by operators, and only the operators know their practical needs during the operation. As a consequence, in this research work, a new original case study of deep learning image recognition of an engine room simulator will let us develop a user-friendly methodology that lets marine engineers create their own alarms in the engine room based on their own experience and particular needs without any kind of modification of the control panel of the engine room [27]. This automatic sensor warns the few workers in the engine room in case of an alarm or when a critical combination of alarms happens.

What is more, due to the similarities between the power stations employed on land (nuclear power stations, hydroelectric power stations, wind farms and power plants) and the ones employed on board, the methodology proposed in this research work can be easily extrapolated for any type of ship power station and in the land control system. In particular, the Convolutional Neural Network method shows other advantages like resolving the computation real-time performance, which is very important for alarm systems. As a consequence, this new advantage must be analyzed in the future.

## 2. Materials and Methods

The TRANSAS (Leningrad (USSR)) [7] simulator is based on this initial simulator developed by Stefan Kluj [2,28] and is currently the most common certified simulator to train crew members of merchant ships. Despite this, this teaching knowledge and procedure can be employed in other well-known simulators like that employed in the aerospace industry and power stations. The main intention is to train the workers with simulators that emulate real power stations, letting them understand new systems and the relation between systems and between systems and equipment. As explained before, for the particular case of merchant ships’ engine room simulators, this learning methodology is compulsory to certify students in certain knowledge.

### Virtual Engine Room Systems and Control Panel

**Figure 1:**
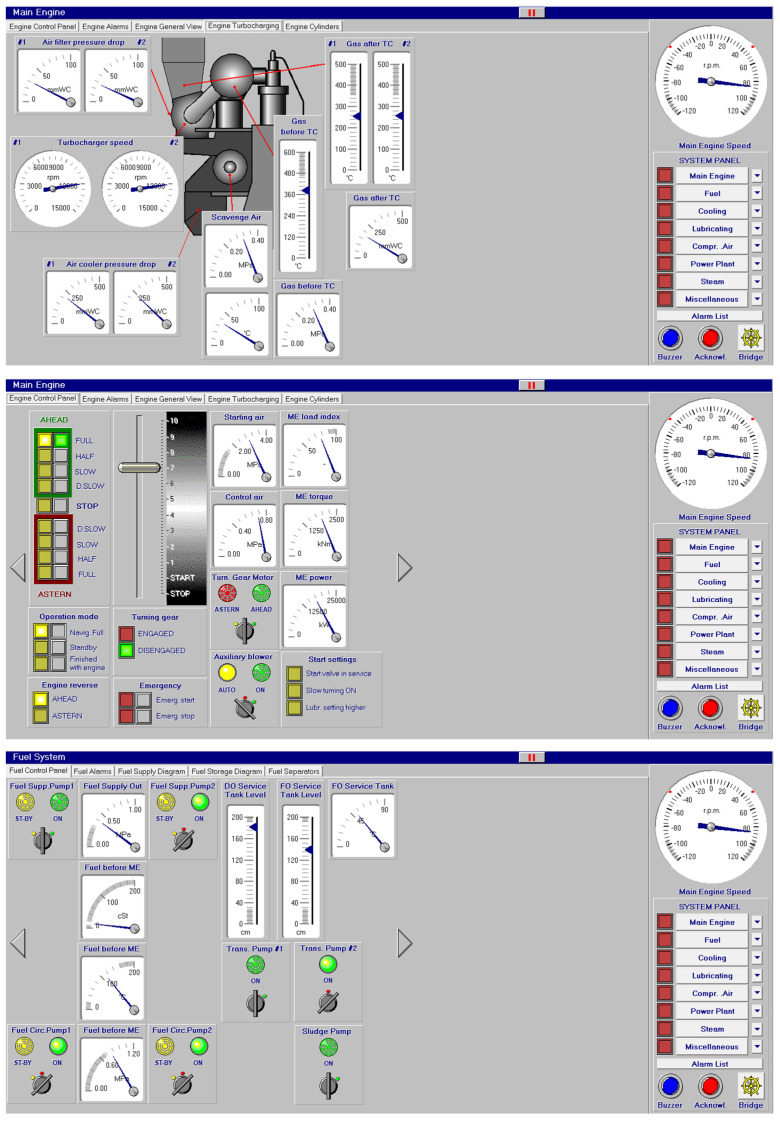
Main engine and control panel.

As mentioned before, each of the systems of a merchant ship is controlled from the control engine room. In this sense, the main engine system (temperatures…) and its controls are represented in Figure 1 with the telegraph, buttons to start pumps of fuel and diesel oil, blowers and turning gear. All these elements are controlled from this screen.

**Figure 2:**
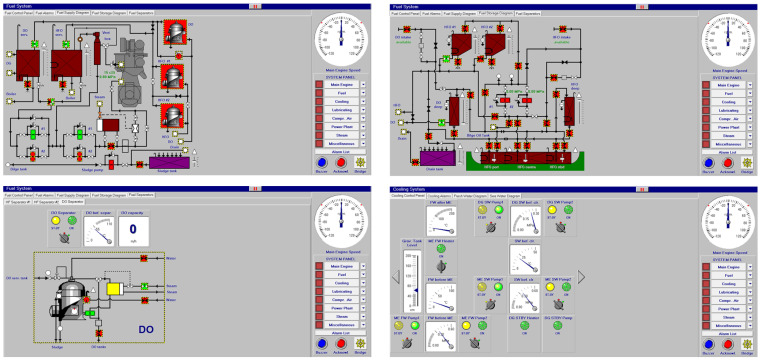
Fuel oil/diesel oil system and control panel.

Figure 2 represents its associated fuel oil/diesel oil system with each tank, valve and purifier. All these elements are supervised and controlled from the figures shown below.

**Figure 3:**
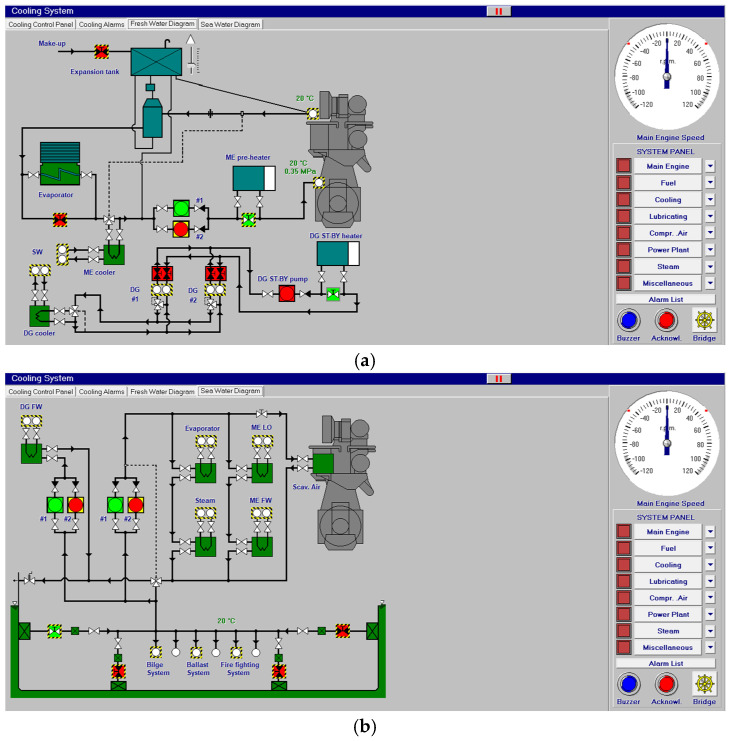
Fresh water (**a**) and sea water system (**b**).

At the same time, in Figure 3, fresh water and saltwater systems are controlled on their visual screen by clicking over the valves to be opened or closed.

**Figure 4:**
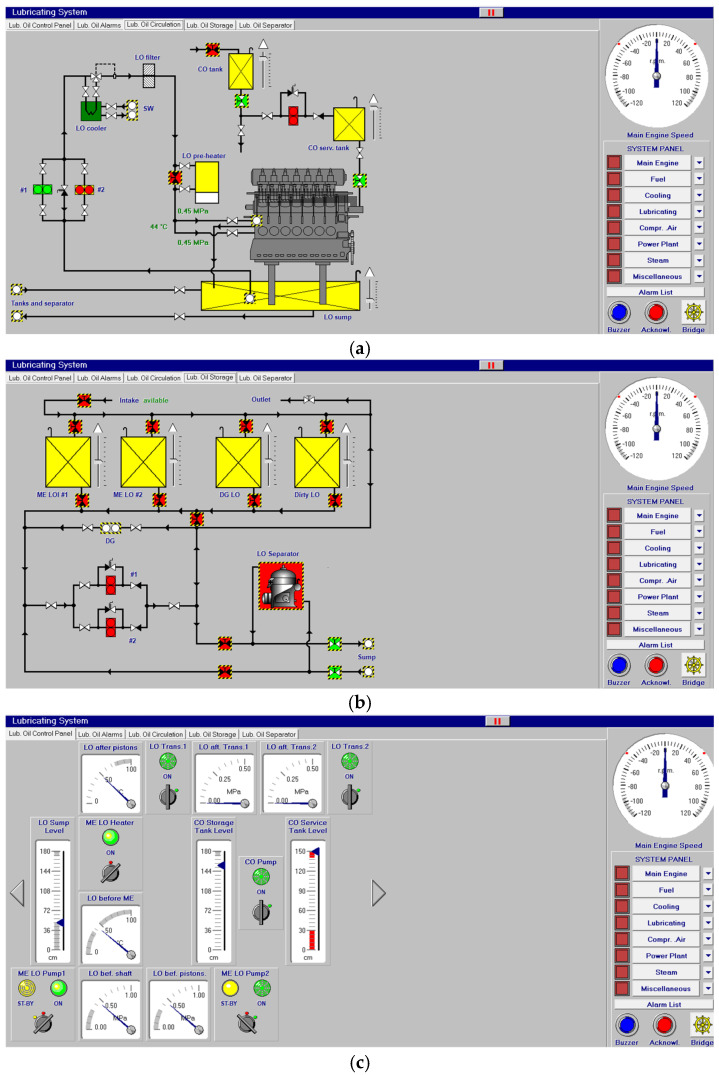
Lubricating oil system (**a**,**b**) and control panel (**c**).

Figure 4 shows the lubricating oil system where two types of oils (oils for cylinders and oils for general lubricating) are fed to the main engine after heaters and coolers, which, at the same time, are fed with the aforementioned fresh water and salt water systems. On the right of Figure 4, a purifier can be seen that, in a closed loop, treats the oil to be stored in the tanks with several pumps controlled from the control system screen shown below.

**Figure 5:**
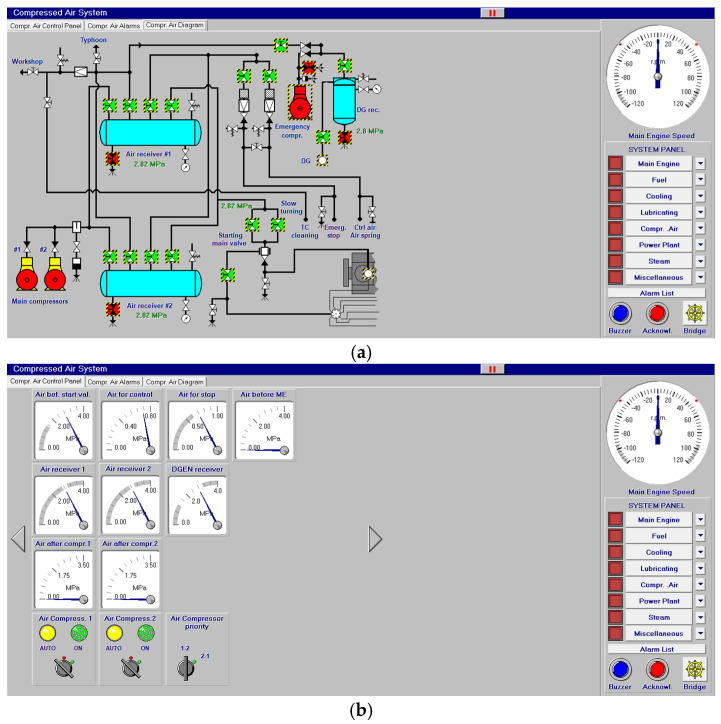
Compressed air system (**a**) and its control panel (**b**).

**Figure 6:**
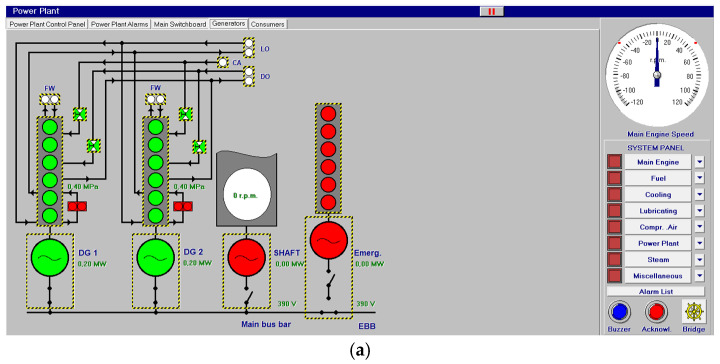

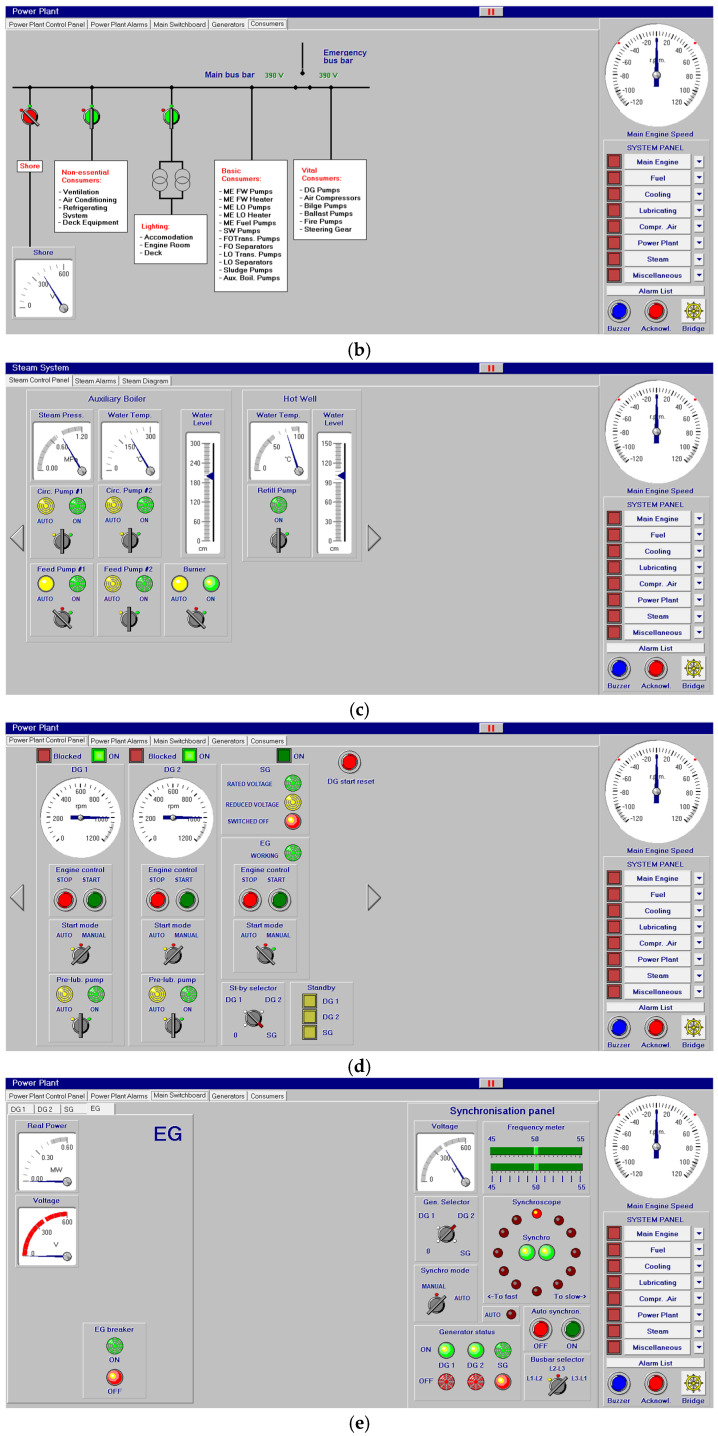
Electrical system (**a**,**b**) and control panel (**c**–**e**).

**Figure 7:**
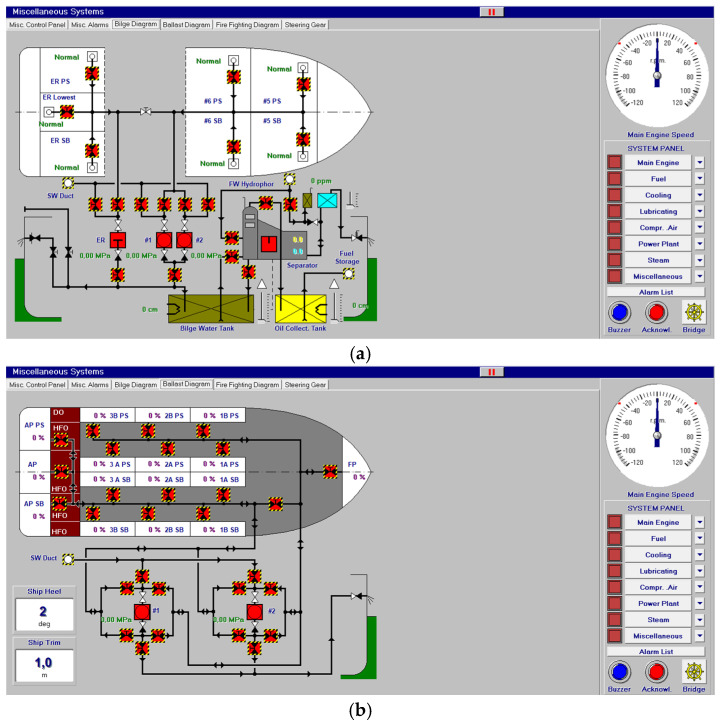
Bilge (**a**) and ballast water system (**b**).

**Figure 8:**
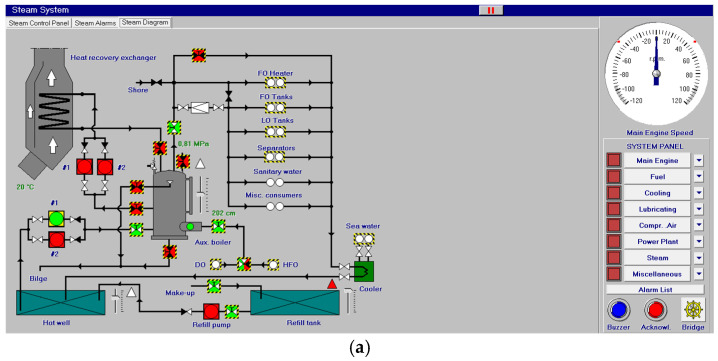

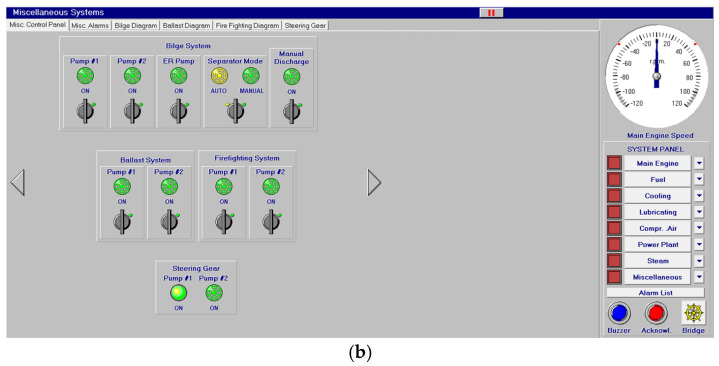
Steam diagram (**a**) and control panel (**b**).

Other systems, like compressed air, electrical, bilge and water ballast and steam, with their associated control systems, are shown in Figure 5, Figure 6, Figure 7 and Figure 8. All of them are realistic systems and controls employed on board that must be supervised by one marine engineer officer.

**Figure 9:**
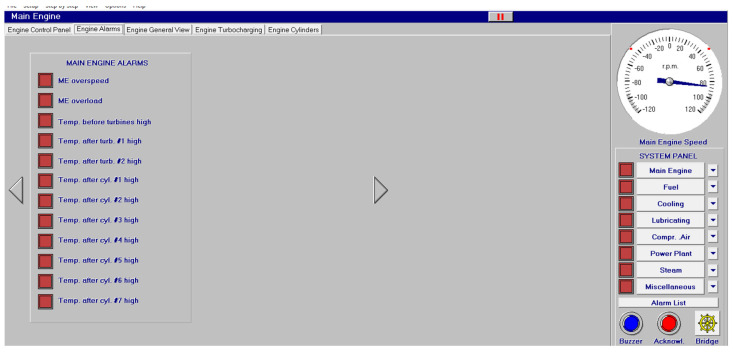
Main engine alarms (12 alarms).

**Figure 10:**
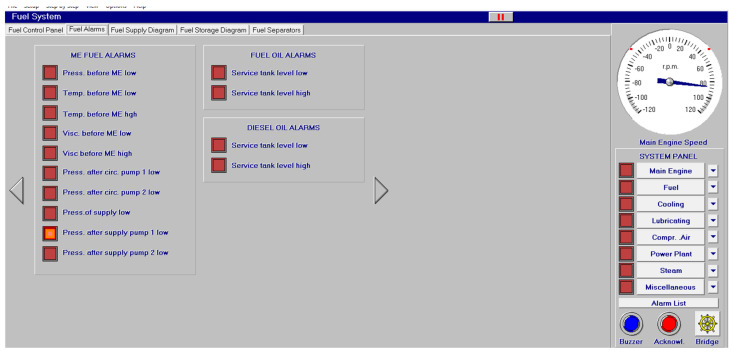
Fuel alarms (14 alarms).

**Figure 11:**
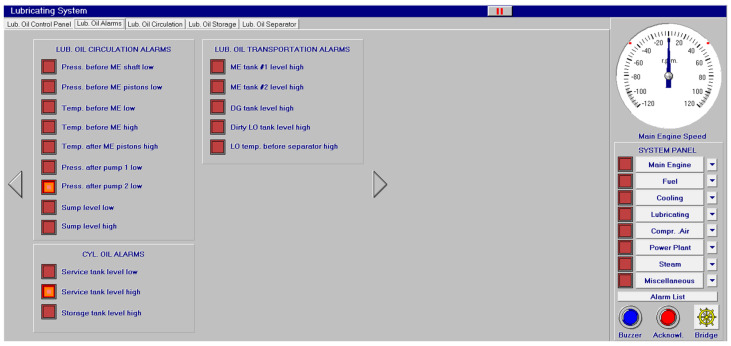
Lubricating oil alarms (17 alarms).

**Figure 12:**
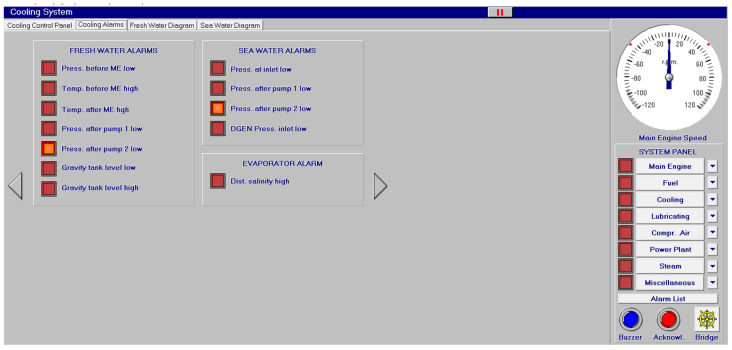
Cooling alarms (12 alarms).

**Figure 13:**
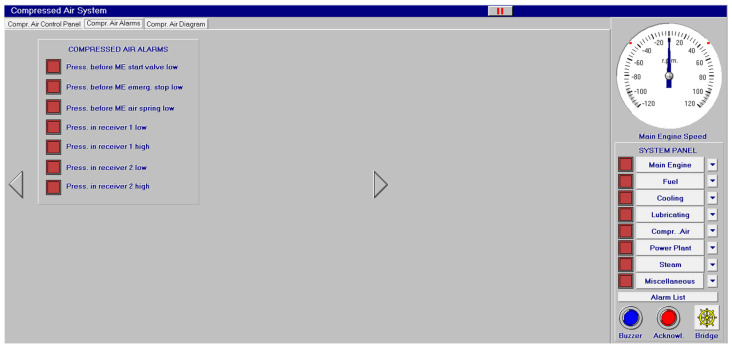
Compressed air alarms (7 alarms).

**Figure 14:**
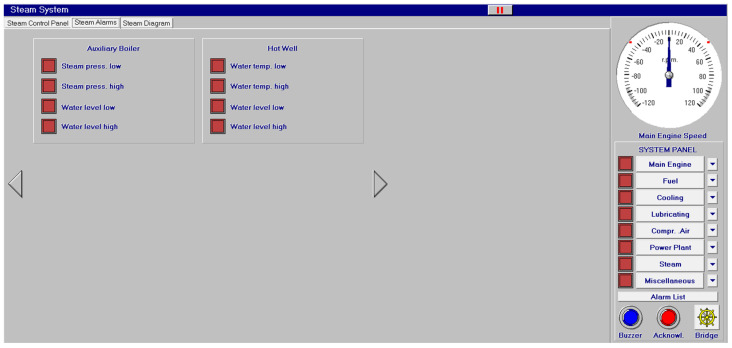
Steam alarms (8 alarms).

**Figure 15:**
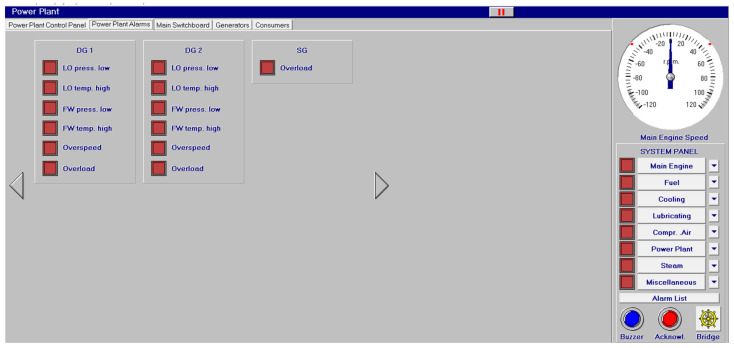
Electrical system (13 alarms).

**Figure 16:**
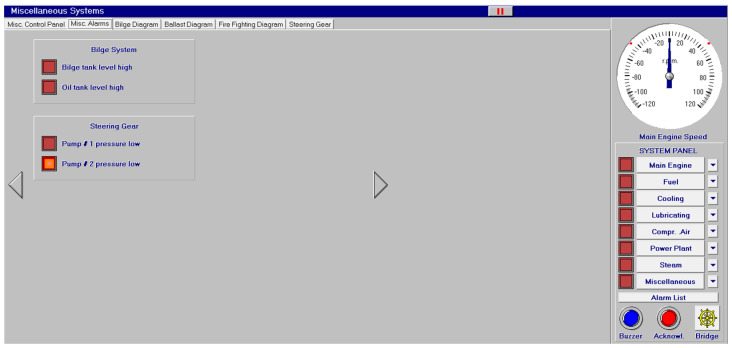
Miscellaneous alarms (4 alarms).

Different screen alarms are employed during the control of a power station in its operations, and only the most relevant alarms are reinforced with acoustic signals and lights. In this sense, Figure 9, Figure 10, Figure 11, Figure 12, Figure 13, Figure 14, Figure 15 and Figure 16 show each alarm associated with each system: main engine, fuel, lubricating, cooling, compressed air, steam, electrical and other miscellaneous systems. It is interesting to highlight that not all the alarms have the same relevance. In this sense, on the one hand, the alarms associated with the main engine are of special interest due to the possibility that the alarm could remain on for some minutes; the main engine will be stopped and the ship will experience a blackout (no lights and no propulsion system), which could clearly be a risky situation in port operations, for instance. On the other hand, an alarm indicating a low level of fuel in tanks is not of particular relevance due to the fact that there are other parallel tanks and the main engine can be fed for hours after its associated alarm is shown. As a consequence, the combination of some alarms may be of critical interest and cannot be usually recognized due to the high number of alarms per working day on board.

As a consequence, during a normal port-stating process of the power plant, more than 90 alarms must be supervised by the marine engineer officer who, at the same time, is manually operating other remaining system elements and supervising its state. What is more, it must be highlighted that the engine room is controlled by three marine engineers, most of the time a chief engineer, an officer and an engine cadet. As a consequence, there are too many alarms that are associated with the operation of each system, which, at the same time, must be associated with an incorrect operation of the other systems. For instance, an excessive temperature before the turbines of the main engine (Figure 9) can be associated with a incorrect temperature or reduced flow of the fresh water system (Figure 3a), which, at the same time, is associated with an incorrect flow or temperature of the salt water system (Figure 3b). This example shows a daily situation that demonstrates the complex work carried out in noisy spaces with temperatures over 40 degrees centigrade. These conditions reduce the concentration of the crew members and enhance the probability of errors. As a consequence, the main objective of this work is to develop a methodology to identify some particular combination of alarms that are critical for common working activities by an artificial intelligence procedure based on image recognition (deep learning) that may be of help to the digital twin of a ship and that can be configured in a few days in every ship. This can reduce the risk of accidents by guiding the marine engineers in their daily operations.

## 3. Results and Discussion

As mentioned previously, the main intention of this work is to develop an artificial intelligence procedure to be employed in ships and power stations that may reduce the risks of accidents by guiding the marine engineering officer in their activities and, in particular, by understanding the combination of alarms and their relevance in real time. To do so, some of the most relevant alarms that can be observed during navigation or in port operations were recorded with the computer image recorder of the control system. As an alternative procedure, an external camera pointing to the monitor of the control system is proposed. At the same time, the images associated with normal operation without alarms are taken as a reference for a deep learning neural network training process. In particular, no learning transfer procedure was selected due to the need for an exact adjustment to the illumination of each alarm with respect to the non-illumination condition.

**Figure 17:**
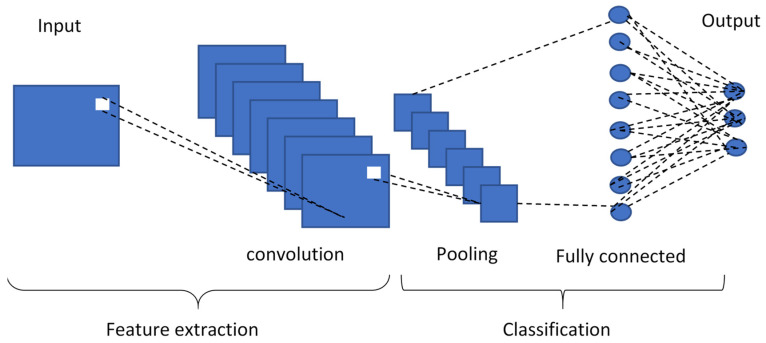
Structure of a CNN.

As a type of feed-forward neural network, a convolutional neural network will recognize images by itself in accordance with a filter (kernel), with the structure described in two sections shown in Figure 17. The first section employs an image input layer and three convolution 2D layers. The second section is a fully connected layer (which utilizes the output from the convolution process and predicts the class of the image), with a softmax layer employed for 70 epochs to ensure an adequate convergence between training and validation accuracy results.

The hyperparameters employed were a learning rate of 0.01, maximum epoch to convergence of 4 and shuffle every epoch and a validation frequency of 30. The optimization algorithm employed in this neural network was stochastic gradient descent with momentum (sgdm), which will update the network parameters in each loop.

The number of training files was modified in accordance with the accuracy obtained and the complexity of the image recognition. In this sense, for initial experiments (Section 3.1), 80 images were employed, while for the image identification of each particular alarm (Section 3.2), 260 images were employed. At the same time, the image quality was 720 × 128, which is a reduced resolution, to reinforce the adequacy of this procedure with low-resolution images.

### 3.1. OK and NOT OK Test

**Figure 18:**
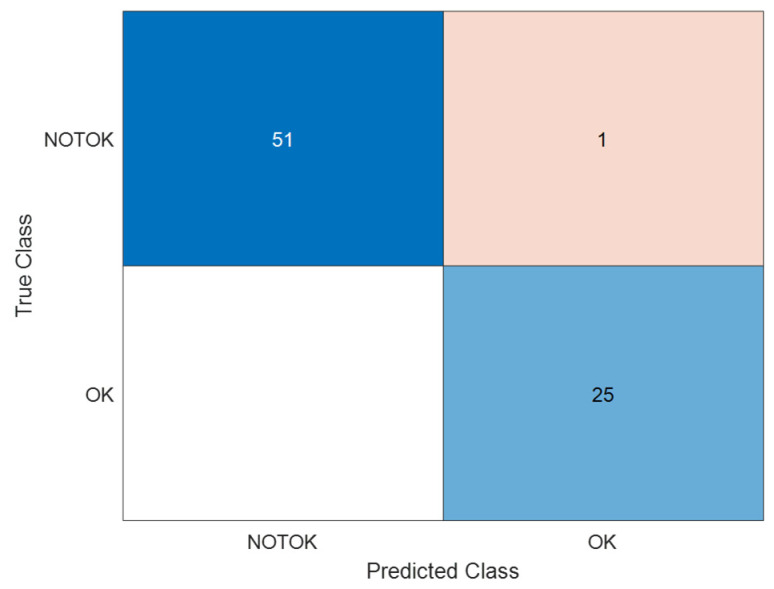
Confusion matrix of the initial classification of images (OK/NOT OK).

The first initial test of the proposed control system was focused on detecting the normal working condition of the engine room (no alarms) with respect to a situation where any alarms are activated. In this sense, the type of alarm is not identified, and a simple warning is issued to marine engineering officers through the messages OK (no alarms) and NOT OK (alarms activated). As mentioned before, 80 images with and without alarms were employed and classified with great accuracy, as shown in Figure 18.

**Figure 19:**
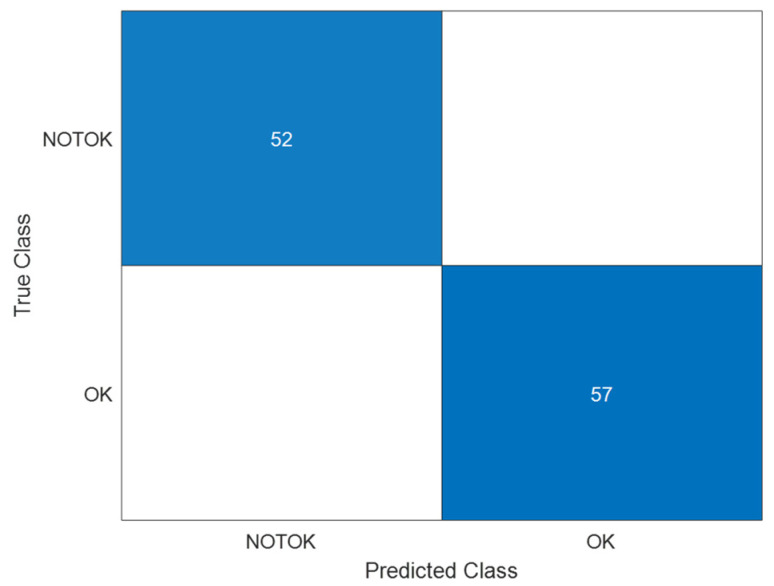
Confusion matrix of the initial classification of images (balanced data R = 1).

**Figure 20:**
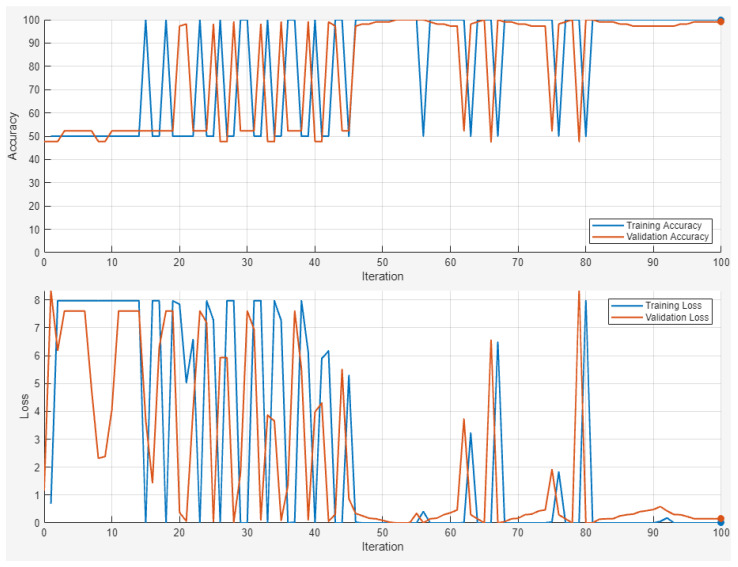
Accuracy and loss of the training and validation processes for each iteration (OK/NOT OK classification).

The confusion matrix of Figure 18 shows that the data employed are imbalanced; therefore, more images were added to the database and the training and classification process was carried out again, as shown in Figure 19. What is more, the epoch number was incremented to 100 epochs to ensure the visual convergence of the training and validation process, as shown in Figure 20.

### 3.2. Identification of Particular Alarms

**Figure 21:**
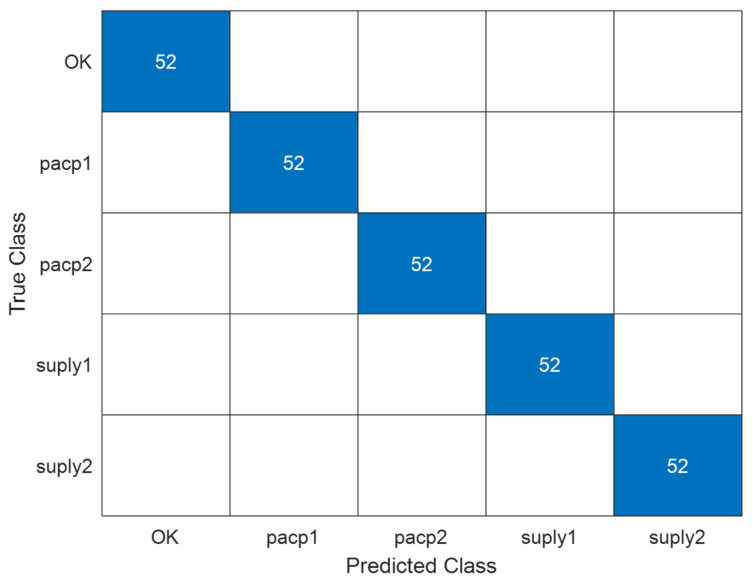
Confusion matrix of the image classification of a normal condition and four types of alarms.

**Figure 22:**
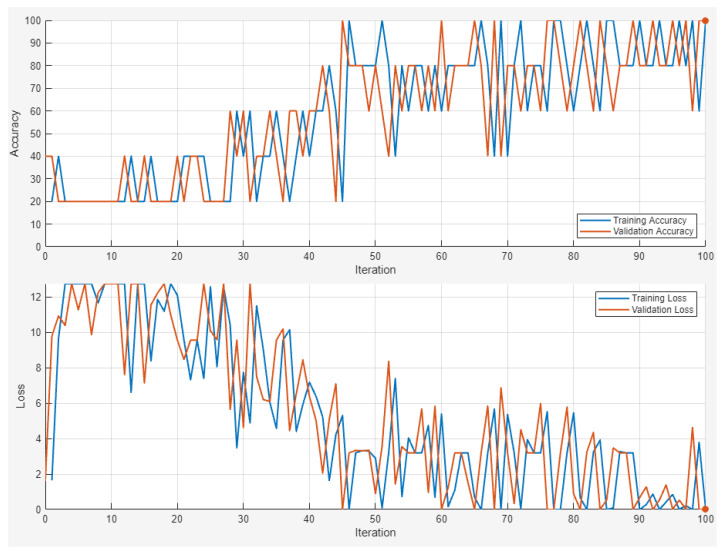
Accuracy and loss of the training and validation processes for each iteration (five classifications).

Based on the previous results, it is of interest to increase the objectives until the identification of each alarm by this same procedure. As a consequence, more images (nearly 300 images) were introduced into the four additional frequency alarms and the OK condition (no alarms) classification was proposed for this neural network. As can be observed in Figure 21, the convolutional neural network reached a perfect classification of images, with convergence after 100 iterations (Figure 22). The alarms selected were abbreviated as OK (no alarms), pressure after circulation pump 1 low (pacp1), pressure after circulation pump 2 low (pacp2), pressure after supply pump 1 (suply1) and pressure after supply pump 2 (suply2).

### 3.3. Illustrative Test of the Use of the Trained CNN

**Figure 23:**
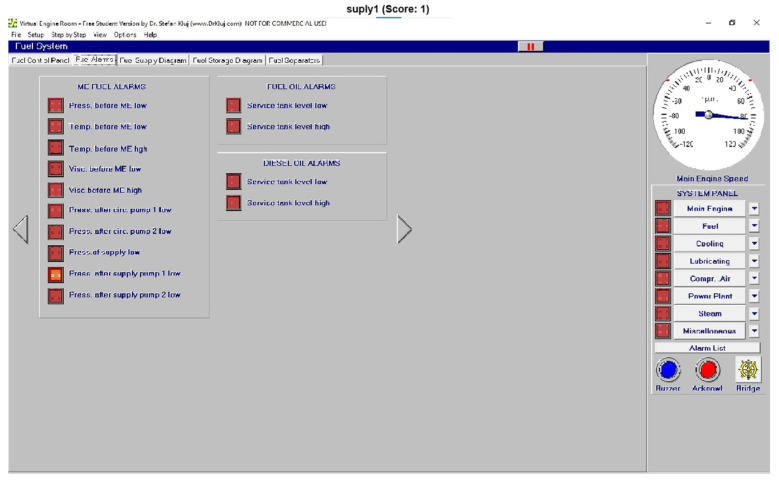
Test of the pressure after supply pump 1.

**Figure 24:**
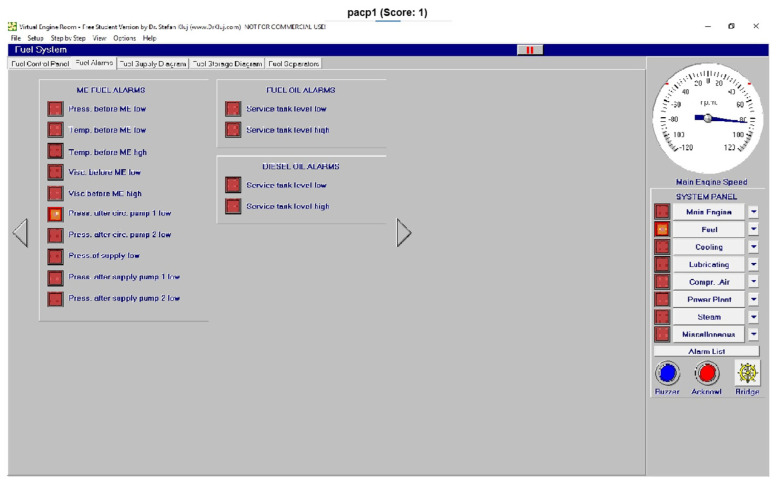
Test of the pressure after circulation pump 2 low (pacp2).

**Figure 25:**
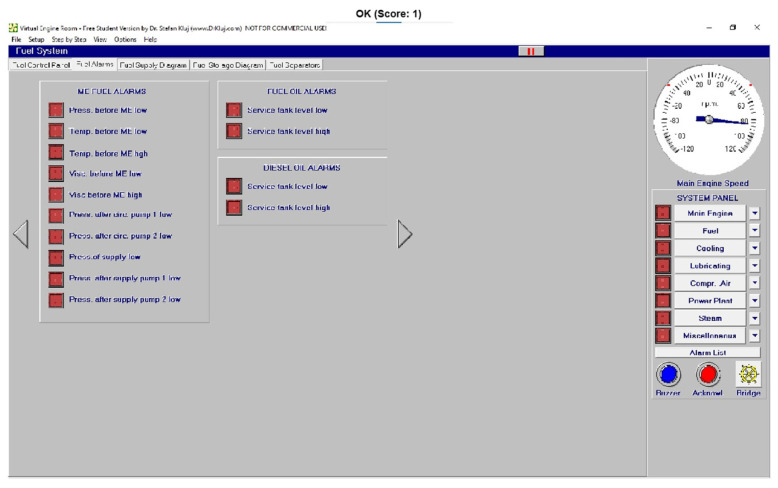
Test of the no alarms condition (OK).

Once the general identification of each image was achieved, some examples, as a test of this technology, are shown in this section. As can be observed, with an accuracy of 100%, each alarm was identified with images with a lower resolution than that captured on screen or by any external camera to reduce the weight of the calculation procedure, as reflected in Figure 23, Figure 24 and Figure 25. 

In this sense, the pressure after supply pump 1 is shown and clearly identified in Figure 23, and Figure 24 shows the identification of the pressure after circulation pump 2 low (pacp2). Finally, the identification of no alarms (OK condition) is clearly identified in Figure 25.

As a consequence of such good results, it can be concluded that it is possible to develop an artificial intelligence model by recognizing images that will help the operator to gain a better understanding of a high number of alarms but without removing the control of the process from the marine engineer officer in accordance with international standards. What is more, this technology can be employed in most of the power stations and in most activities where any kind of visual alarm is indicated by a computer, as shown in this case study.

It is of special interest to understand that the accuracy of this training process is elevated with respect to previous studies where slight modification of the objective images will induce an increase of the identification error. In this type of image, the objective function is always the same and this is the origin of the interest in image recognition. What is more, it is evident that a specialized control algorithm of the Supervisory Control and Data Acquisition (SCADA) of the control system may allow us to obtain the same accuracy, but the aim of this case study was to implement new particular algorithms once the control system was implemented, as used in power stations and ships. In this last case, an external camera placed in front of the control system will let us identify alarms without interfering with the ship control system and monitors, which is something of interest when the interests of any kind of marine underwriter are involved. 

Another relevant parameter to be employed in this type of CNN is the need to employ an adequate number of layers; for this type of image, three convolutional 2D layers and a learning rate of 0.01 were adequate.

Finally, more case studies must be developed to increase the identification of more than 90 alarms and situations based on the experience of the workers and optimized CNN [29], which, most of the time, are slightly further from the initial design of the control system made in the shipyard. As a consequence, this tool will help crew members obtain a more efficient and user-friendly knowledge of their working environment, with a consequent reduction in the stress and risks on board.

## 4. Conclusions

Several conclusions can be derived from this research work:

1. It is possible to employ CNN to recognize particular alarms in ships and power stations.
2. The accuracy of this procedure reaches 100%.
3. The optimal configuration of this camera as a sensor was of three convolutional layers and balanced data.
4. This tool will let operators adjust the control system designed in the shipyard to their particular needs by employing their own control system monitors or some external cameras.
5. Future research works must be carried out to improve the quality of the work on board and reduce its related risks.

## Data Availability

Data are contained within the article.

## References

[1] International Maritime Organization (2017). International Convention on Standards of Training, Certification and Watchkeeping for Seafarers.

[2] Kluj S. https://drkluj.com.

[3] International Maritime Organization (2020). Convention for the Safety of Life at Sea, 1974 (SOLAS).

[4] International Maritime Organization (2022). International Convention for the Prevention of Pollution from Ships, 1973 (MARPOL).

[5] Laskowski R., Chybowski L., Gawdzińska K., Rocha A., Correia A., Costanzo S., Reis L. (2015). An Engine Room Simulator as a Tool for Environmental Education of Marine Engineers. New Contributions in Information Systems and Technologies. Advances in Intelligent Systems and Computing.

[6] Seddiedk I.S. (2019). Viability of using engine room simulators for evaluation machinery performance and energy management onboard ships. Int. J. Marit. Eng..

[7] TRANSAS Simulators. https://wms.transas.com.

[8] Virtual Engine Room 7. https://drkluj.com/simulators/virtual-engine-room.

[9] AMOS Maintenance SPECTEC. AMOS Maintenance and Procurement..

[10] Chen R., Zhang J., Shen H. (2024). Research on Ship-Engine-Room-Equipment Detection Based on Deep Learning. J. Mar. Sci. Eng..

[11] Songül Sarıalioğlu S., Uğurlu O., Aydın M., Vardar B., Wang J. (2020). A hybrid model for human-factor analysis of engine-room fires on ships: HFACS-PV&FFTA. Ocean Eng..

[12] Zhang J., Jiang J., Zhang S., Zhang P., Dong J., Sun Z. (2023). A review of research on intelligent engine room systems. Adv. Mach. Mater. Sci. Eng. Appl. IV.

[13] Zou Y., Zhang J., Du T., Jiang X., Wang H., Zhang P., Zhang Y., Sun P. (2023). Smoke Detection of Marine Engine Room Based on a Machine Vision Model (CWC-Yolov5s). J. Mar. Sci. Eng..

[14] Islam R., Khan F., Abbassi R., Garaniya V. (2018). Human error probability assessment during maintenance activities of marine systems. Saf. Health Work.

[15] Chowdhury M.N., Shafi S., Arzaman A.F.M., Teoh B.A., Kadhim K.A., Salamun H., Kadir F.K.A., Said S., Kadir K.A., Embong A.M. (2024). Navigating human factors in maritime safety: A review of risks and improvements in engine rooms of ocean-going vessels. Int. J. Saf. Secur. Eng..

[16] Islam R., Anantharaman M., Khan F., Garaniya V. (2020). A review of human error in marine engine maintenance. Int. J. Mar. Navig. Saf. Sea Transp..

[17] Marchal J.M., Camacho E.F. Design of an intelligent supervisor of a ship engine room. Proceedings of the IFAC Artificial Intelligence in Real-Time Control.

[18] Zhang P., Song Z., Li C., Liu Y., Zou Y., Zhang Y., Sun P. (2024). A study of engine room smoke detection based on proactive machine vision model for intelligent ship. Expert Syst. Appl..

[19] Qi J., Zhang J., Meng Q. (2021). Auxiliary Equipment Detection in Marine Engine Rooms Based on Deep Learning Model. J. Mar. Sci. Eng..

[20] Shang D., Zhang J., Zhou K., Wang T., Qi J. (2022). Research on the Application of Visual Recognition in the Engine Room of Intelligent Ships. Sensors.

[21] Kim S.-D., Bae C.-O. (2023). Unmanned Engine Room Surveillance Using an Autonomous Mobile Robot. J. Mar. Sci. Eng..

[22] CIAIN Reports. https://www.transportes.gob.es/organos-colegiados/ciaim.

[23] Maceiras C., Pérez-Canosa J.M., Vergara D., Orosa J.A. (2021). A Detailed Identification of Classificatory Variables in Ship Accidents: A Spanish Case Study. J. Mar. Sci. Eng..

[24] Zou Y., Zhang Y., Ma Z. (2021). Emergency Situation Safety Evaluation of Marine Ship Collision Accident Based on Extension Cloud Model. J. Mar. Sci. Eng..

[25] Pérez-Canosa J.M., Orosa J.A. (2024). Proposal of a New Control System Making Use of AI Tools to Predict a Ship’s Behaviour When Approaching the Synchronism Phenomenon. Appl. Sci..

[26] Xiong C., Hao Shi H., Li J., Wu X., Gao R. (2024). Informer-Based Model for Long-Term Ship Trajectory Prediction. J. Mar. Sci. Eng..

[27] Chon H., Oh D., Noh J. (2023). Classification of Hull Blocks of Ships Using CNN with Multi-View Image Set from 3D CAD Data. J. Mar. Sci. Eng..

[28] Orosa García J.A., Bouzón R., Pita M., Kluj S. (2024). Ship’s Services: Machine Simulator (2t Main Engine).

[29] Ang K.M., El-Kenawy E.-S.M., Abdelhamid A.A., Ibrahim A., Alharbi A.H., Khafaga D.S., Tiang S.S., Lim W.H. (2022). Optimal Design of Convolutional Neural Network Architectures Using Teaching–Learning-Based Optimization for Image Classification. Symmetry.

